# Retroperitoneoscopic radical nephrectomy with a small incision for renal cell carcinoma: Comparison with the conventional method

**DOI:** 10.1186/1477-5751-10-11

**Published:** 2011-08-16

**Authors:** Hiroki Ito, Kazuhide Makiyama, Takashi Kawahara, Futoshi Sano, Takayuki Murakami, Narihiko Hayashi, Yasuhide Miyoshi, Noboru Nakaigawa, Masahiro Yao, Yoshinobu Kubota

**Affiliations:** 1 Department of Urology, Yokohama City University Graduate School of Medicine and School of Medicine, Yokohama, Japan

**Keywords:** the retroperitoneoscopic radical nephrectomy method with a small incision, surgical outcome

## Abstract

**purpose:**

When retroperitoneoscopic radical nephrectomy for renal cell carcinoma was introduced into our institution, we performed a combined small skin incision method. In this method, a small incision was made to approach the retroperitoneal space prior to setting trockers and thereafter a LAPDISC was placed in the incision to start the retroperitoneoscopic procedure. In this study, we compared the outcomes between the combined small skin incision method ("A method" hereinafter) and the conventional method ("B method" hereinafter).

**material and methods:**

Among the cases of T1N0M0 suspicious renal cell carcinoma treated at Yokohama City University between May 2003 and June 2009, the A method was performed in 51 cases and the B method was performed in 33 cases. The factors in the outcomes compared between the A and B methods were the duration of procedure, volume of bleeding, volume of transfusion, weight of the specimen, incidence of peritoneal injury, rate of conversion to open surgery, and perioperative complications.

**results:**

The duration of the procedure was 214.4 ± 46.9 minutes in the A method group and 208.1 ± 36.4 minutes in the B method group (p = 0.518). The volume of bleeding and the weight of the specimen were 105.5 ± 283.2 ml and 335.1 ± 137.4 g in the A method group and 44.8 ± 116 ml (p = 0.247) and 309.2 ± 126 g (p = 0.385) in the B method group. There was no significant difference in all factors analyzed.

**conclusion:**

The A method would be highly possible to produce stable results, even during the introduction period when the staff and the institution are still unfamiliar with the retroperitoneoscopic surgery.

## Introduction

The technical progress in laparoscopic surgery for renal cell carcinoma has been remarkable. Many institutions have introduced laparoscopic radical nephrectomy for renal cell carcinoma and even retroperitoneoscopic radical nephrectomy for renal cell carcinoma [1]. In recent years, these surgical methods are in widespread use, and the number of reports [2,3] about complications associated with surgery is rising. It has become important to identify how such a surgery can be completed in a safe manner during the introduction period when institutions and staff are still unfamiliar with these surgical methods.

When retroperitoneoscopic radical nephrectomy for renal cell carcinoma was introduced into our institution, we performed a combined small skin incision method in our hospital. That's because we thought that the combined small skin incision method was safer than the conventional method that all procedures were performed with laparoscopic instruments. In this study, we compared the outcomes between the combined small skin incision method ("A method" hereinafter) and the conventional method ("B method" hereinafter).

## Material and methods

**Figure 1:**
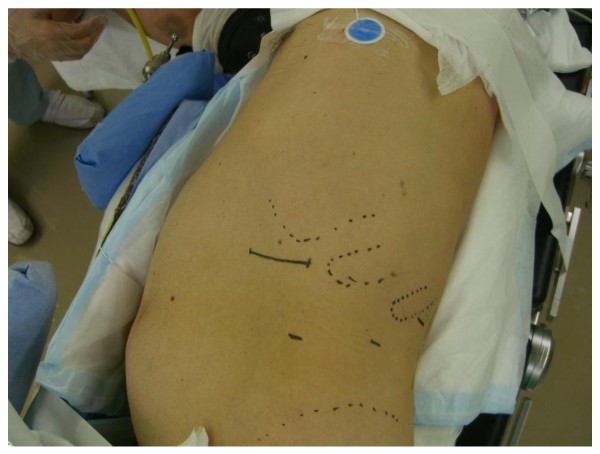
**The surgical procedure of the A method (No.1)**. The patient's position is lateral position and an incision of about 7 cm is made from the top end of the 11^th ^rib to the border of the rectus abdominis.

**Figure 2:**
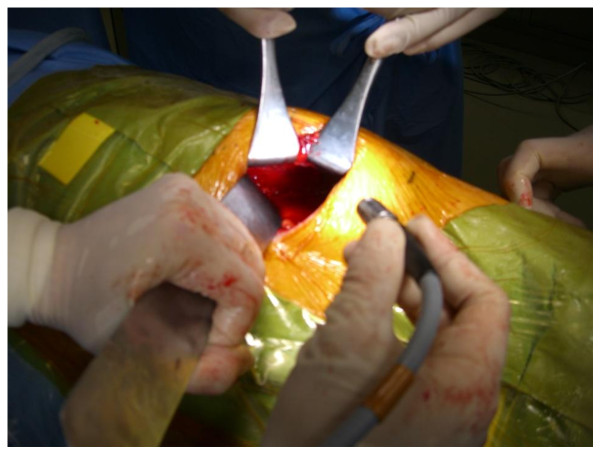
**The surgical procedure of the A method (No.2)**. It is possible to remove the flank pad and to access the retroperitoneal space under one's direct vision.

**Figure 3:**
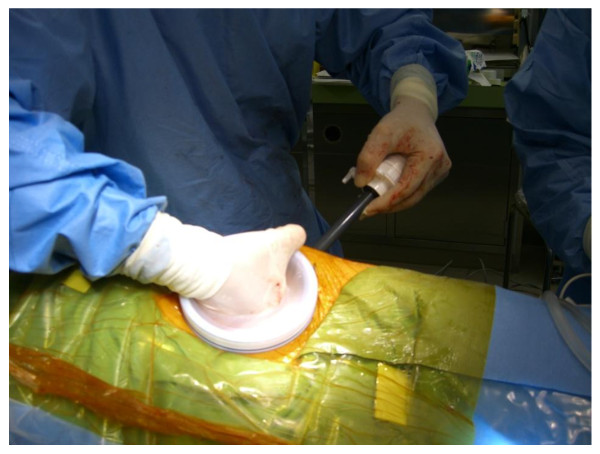
**The surgical procedure of the A method (No.3)**. The LAPDISC is placed in the incision to insert the port.

**Figure 4:**
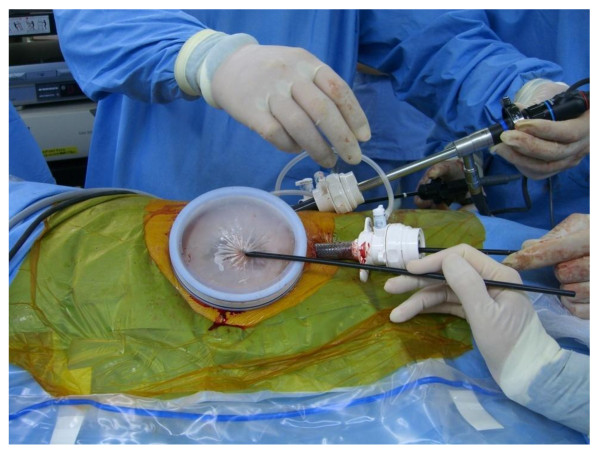
**The surgical procedure of the A method (No.4)**. It is possible to insert forceps through the closed LAPDISC as well as to control and operate them.

The surgical procedure of the A method is shown below. In a lateral position, a lumbar oblique incision 7 cm long is made from the top end of the 11^th ^rib to the border of the rectus abdominis to approach the retroperitoneal space (Figure 1). Under direct vision, the flank pad was removed through the skin incision with instruments, not with hands (Figure 2). Subsequently, the ureter and Gerota's fascia were dissected from the peritoneum. After the dissection, hand port device, the LAPDISC(120 × 120 mm, Hakko, Osaka, Japan), was attached to the skin and three 12 mm trocars were placed (Figure 3). The subsequent procedures are performed by retroperitoneoscopic surgery with carbon dioxide insufflations when the LAPDISC was cloesd. It was possible to insert forceps through the LAPDISC as well as to control and operate them (Figure 4). The isolated kidney was removed through the skin incision [4,5].

Among the cases of suspicious T1N0M0 renal cell carcinoma treated at Yokohama City University between May 2003 and June 2009, the A method was performed in 51 cases (preoperative diagnosis was T1a in 30 cases and T1b in 21 cases) and the B method was performed in 33 cases (preoperative diagnosis was T1a in 20 cases and T1b in 13 cases). The A method was performed during the period between May 2003 and January 2008 and the B method was performed during the period between September 2007 and June 2009. The period between September 2007 and January 2008 was a period of transition from the A method to the B method. During this period, the A method was performed in 8 cases while the B method was performed in 5 cases. A total of six surgeons handled these cases, and the A method was employed by six surgeons while the B method was employed by four surgeons.

According to the criteria applied in our hospital for selecting the surgical method for renal cell carcinoma, the main indication for retroperitoneoscopic radical nephrectomy for renal cell carcinoma is T1N0M0 renal cell carcinoma. That's because we thought that laparoscopic approach caused peritoneal adhesion more than retroperitoneoscopic approach. In T2-T3aN0M0 renal cell carcinoma as well as in T1 tumours located dorsally, a laparoscopic approach is preferred. Furthermore, it has been reported recently that partial nephrectomy for renal cell carcinoma exhibits higher efficiency [6], and thus open partial nephrectomy or laparoscopic partial nephrectomy is more frequently performed in the treatment of T1a renal cell carcinoma in our hospital [7].

The factors in the outcomes compared between the A and B methods were the duration of procedure, volume of bleeding, volume of transfusion, weight of the specimen, incidence of peritoneal injury, rate of conversion to laparotomy, and perioperative complications. The Student's *t-*test and the Fisher's exact test were used in the analysis.

## Results

The patients' backgrounds are as described in Table 1. The average weight and the average BMI in the A method cases were 64.6 ± 14.6 kg and 24.3 ± 3.85, whereas in the B method cases, these were 58.1 ± 12.2 kg (p = 0.0395) and 21.9 ± 3.58 (p = 0.0055). There was a difference observed in patients' backgrounds between these two groups. However, because the sample cases had been selected without any intentional action at the time, it was believed that the difference was accidental. There was no difference observed in the other factors (age, male-to-female ratio, body height, and bilateral difference in tumor location).

**Table 1:** demographic and tumor data

	A method	B method	p-Value
No. pts	51	33	
mean age ± SD	62.55 ± 12.22	60.88 ± 14.46	0.571 *
male-to-female ratio(male/female)	34cases/17cases	22cases/11cases	>0.999 *
mean body height ± SD (cm)	162.7 ± 8.9	162.8 ± 11.1	0.952 **
mean body weight ± SD (kg)	64.6 ± 14.6	58.1 ± 12.2	0.0395 **
mean BMI ± SD	24.3 ± 3.85	21.9 ± 3.58	0.0055 **
preoperative tumor diameter ± SD(mm)	40.5 ± 12.6	40 ± 11.7	0.861 *
laterality (left/right)	26/25	16/17	>0.999 *

* Student's t test ** Fisher's exact test.

Tumor characteristics of all 84 cases are indicated in Table 2. In the A method, preoperative tumor stage was T1a in 30 cases and T1b in 21 cases. In the B method, preoperative tumor stage was T1a in 20 cases and T1b in 13 cases. In the former, pathological tumor stage was T1a in 37 cases, T1b in 9 cases and larger T2 in 5 cases. In the latter, pathological tumor stage was T1a in 25 cases, T1b in 5 cases and larger T2 in 3 cases.

**Table 2:** clinical T stage and pahological data

		A method	B method
preoperative clinical T stage	T1a	30 (59%)	20 (61%)
	T1b	21 (41%)	13 (39%)
pathological classification	clear cell	43 (84%)	26 (79%)
	Papillary	3 (6%)	0
	Chromophobe	2 (4%)	3 (9%)
	benign	2 (4%)	2 (6%)
	Other	1 (2%)	2 (6%)
grade	G1	15 (29%)	8 (24%)
	G2	26 (51%)	20 (61%)
	G3	7 (14%)	1 (3%)
	Unclear	3 (6%)	4 (12%)
microscopic venous invasion	Negative	38 (75%)	18 (55%)
	Positive	13 (26%)	11 (33%)
	Unclear	0	4 (12%)
pathological T stage	T1a	37 (73%)	25 (76%)
	T1b	9 (18%)	5 (15%)
	T2-	5 (10%)	3 (9%)

* Student's t test ** Fisher's exact test.

Histological data and nuclear grade was available. In the A method, 84.3% had clear cell tumors, and 15.7% had other histologies, on the other hand, 78.7% had clear cell tumors, and 21.3% had other histologies in the B method. In the former, Pathological nuclear grade was 1 in 29.4%, 2 in 51%, 3 in 13.7%, and in the latter, that grade was 1 in 24.2%, 2 in 60.6%, 3 in 3%.

In the A method group, complications were found in 4 cases: a descending mesocolon injury in 1 case, a renal vein injury in 1 case, damage to the renal cortex in 1 case, and damage to the inferior surface of the liver in 1 case. In the B method group, complications were found in 1 case: a renal vein injury in 1 case. Open conversion was found in 1 case in each group: the case of damage to the renal cortex in the A method group and the case of renal vein injury in the B method group. Blood transfusion was required in 2 cases in the B method group: 1 case of pre-surgery anemia and 1 case of renal vein injury. Peritoneal injury was identified in 15 cases in the A method group (29.4%) and 8 cases in the B method group (24.2%) (p = 0.803).

The duration of the procedure was 214.4 ± 46.9 minutes in the A method group and 208.1 ± 36.4 minutes in the B method group (p = 0.518). The volume of bleeding and the weight of the specimen were 105.5 ± 283.2 ml and 335.1 ± 137.4 g in the A method group and 44.8 ± 116 ml (p = 0.247) and 309.2 ± 126 g (p = 0.385) in the B method group. There was no significant difference in all factors analyzed (Table 3).

**Table 3:** intraoperative and postoperative parameters between A method and B method

	A method	B method	p-Value
mean operative time ± SD (min)	214.4 ± 46.9	208.1 ± 36.4	0.518 **
blood loss ± SD (ml)	105.5 ± 283.2	44.8 ± 116	0.247 **
blood transfusion ± SD (unit)	0	0.242 ± 1.09	0.115 **
weight of specimens ± SD (g)	335.1 ± 137.4	309.2 ± 126	0.385 **
peritoneal injury	15cases (29.4%)	8cases (24.2%)	0.803 *
complications	4cases (7.8%)	1cases (3.03%)	0.644 *
open conversion	1cases (1.96%)	1cases (3.03%)	>0.999 *

* Student's t test ** Fisher's exact test.

## Discussion

Retroperitoneoscopic surgery must be performed in a narrow operative space and is thus said to be technically difficult to perform, and therefore laparoscopic surgery was recommended in the past [8]. However, the invention of atraumatic balloon dilation has made it easier to secure a space [8], and retroperitoneoscopic surgery has been in widespread use in recent years [1]. Many reports have presented results of a comparison of retroperitoneoscopic surgery with open surgery and laparoscopic surgery, and the studies concluded that retroperitoneoscopic radical nephrectomy is in no way inferior to other traditional surgical methods [9,10]. It is certain that there have been significant technical improvements in retroperitoneoscopic surgery. At the same time, there are also reports on the existence of learning curves [8]. Tobias-Machado M et. al. [11] reported that a clear learning curve was obtained in the initial 15 cases.

Inderbir S. Gill et. al. [12] compared the outcomes of a laparoscopic partial nephrectomy for renal cell carcinoma soon after introducing it into their institution with the outcomes of an open laparoscopic partial nephrectomy for renal cell carcinoma, and reported that the incidence of intraoperative complications was significantly higher in laparoscopic surgery. This indicates that laparoscopic partial nephrectomy for renal cell carcinoma is technically difficult to perform. Furtheremore, however laparoscopic surgery is said to be safer, that may also induce complications. The complications tend to be occurred particularly when laparoscopic surgery is performed during the introduction period when surgeons and medical staff are still unfamiliar with this surgical method and when learning curves have not yet been determined.

Safer laparoscopic surgical methods, such as hand-assisted laparoscopic surgery, have now been devised [13] and it has been proved that these methods are less invasive than open surgery. The A method was similar to this hand-assisted laparoscopic surgery. But the A method could be better than hond-assited method in terms of being able to using abdominal air pressure to prevent bleedeing. The A method was performed at the time of introduction of retroperitoneoscopic surgery into our institution and it was possible to obtain stable results.

One of the advantages of the A method could be safety. Since it is possible to remove the flank pad and to access the retroperitoneal space under one's direct vision, it is easier to understand the orientation, and it is possible to more promptly facilitate open conversion in cases of massive bleeding. However, the open conversion in 1 case of the A method was not emergent but selective. So, we were fortunately not given an opportunity to perform it in order to stop massive bleeding.

On the other hand, one of the disadvantages of the A method is larger size of the incision. The incision, which is about 7 cm, is larger than the approximately 5 cm incision in the traditional methods, and the preceding incision limits port sites. In particular, limits of port sites were more prominent with thinner patients and disturbed the surgical procedures in quite a few cases.

In this study, there was no difference in surgical outcomes between the A method and the B method for renal cell carcinoma. Since there was no difference in the outcomes between the A method at the time of introduction of retroperitoneoscopic surgery and the B method when the staff had become familiar with retroperitoneoscopic surgery, it was indicated that the A method could be safer. In addition, we had to state that we have limitations in this study because of small sample size included. Such a small number of sample included in this study might be related to a lack of statistical power. That could induce no difference between the two groups in surgical outcomes.

## Conclusion

This study identified no large differences in the outcomes between the retroperitoneoscopic radical nephrectomy method with a small incision for renal cell carcinoma and the standard method.

It would be highly possible to produce stable results, even during the introduction period when the staff and the institution are still unfamiliar with the retroperitoneoscopic surgery, by applying the procedure of a combined small skin incision.

## Abbreviations

A method: Retroperitoneoscopic radical nephrectomy with a small incision; B method: Standard way of retroperitoneoscopic radical nephrectomy.

## Competing interests

The authors declare that they have no competing interests.

## Authors' contributions

All authors participated in the design and conduct of the study. All authors reviewed and approved the final version of the manuscript.
